# Task-Incremental Learning for Drone Pilot Identification Scheme

**DOI:** 10.3390/s23135981

**Published:** 2023-06-27

**Authors:** Liyao Han, Xiangping Zhong, Yanning Zhang

**Affiliations:** School of Cybersecurity, Northwestern Polytechnical University, Xi’an 710072, China; zxp6329@163.com (X.Z.); ynzhang@nwpu.edu.cn (Y.Z.)

**Keywords:** drone pilot identification, Internet of Things, UAV security, incremental learning, deep learning

## Abstract

With the maturity of Unmanned Aerial Vehicle (UAV) technology and the development of Industrial Internet of Things, drones have become an indispensable part of intelligent transportation systems. Due to the absence of an effective identification scheme, most commercial drones suffer from impersonation attacks during their flight procedure. Some pioneering works have already attempted to validate the pilot’s legal status at the beginning and during the flight time. However, the off-the-shelf pilot identification scheme can not adapt to the dynamic pilot membership management due to a lack of extensibility. To address this challenge, we propose an incremental learning-based drone pilot identification scheme to protect drones from impersonation attacks. By utilizing the pilot temporal operational behavioral traits, the proposed identification scheme could validate pilot legal status and dynamically adapt newly registered pilots into a well-constructed identification scheme for dynamic pilot membership management. After systemic experiments, the proposed scheme was capable of achieving the best average identification accuracy with 95.71% on P450 and 94.23% on S500. With the number of registered pilots being increased, the proposed scheme still maintains high identification performance for the newly added and the previously registered pilots. Owing to the minimal system overhead, this identification scheme demonstrates high potential to protect drones from impersonation attacks.

## 1. Introduction

With the continuous advancement of the Industrial Internet of Things and 5G technology, drones have been integrated with various emerging technologies, such as Software-Defined Networking and Blockchain, for providing reliable service [1,2]. Owing to their high mobility and operability, a drone can act as an individual switch for traffic forwarding in an SDN-based drone communication network. On the other hand, Blockchain technology has also been applied to drone swarms to keep the transactions between drones and pilots secure, cost-effective and privacy-preserving.

Since drones have become indispensable to intelligent traffic systems, many adversaries try to compromise flying drones for malicious purposes. Currently, there are already some vulnerabilities that have been found in commercial drones. For example, the authors in [3] revealed that GPS spoofing attacks could mislead the flying drones to a manipulated destination. Son et al. in [4] also found that resonance effects could exaggerate the Micro-Electro-Mechanical Systems gyroscope estimation bias, preventing drones from undergoing pre-flight checks. Furthermore, networking-based attacks, such as Denial of Services attack [5] and Man-in-the-Middle attack [6], also pose significant threats to flying drones. Compared with the previously mentioned vulnerabilities, pilot impersonation attacks, where the adversaries try to impersonate the victim pilot bearing compromised credentials and send malicious control instructions to the flying drones, pose severe threats to the drone’s security. As the adversary could obtain drone control privileges without generating faked radio signals, the pilot impersonation attacks are more brutal to detect and nerveless to be prevented.

Currently, there are many works [7,8,9,10] that have investigated the validation of the pilot legal status to protect drones from pilot impersonation attacks. For example, Zhang et al. in [7] proposed the utilization of a one-way hash function and bitwise XOR operations for authentication and key agreement at the beginning of the flight. After analyzing with the security tools, they have proven safety under the random oracle model and can achieve the security requirements of the Internet of drones environment to withstand various attacks. After that, Alladi et al. in [8] proposed a Physical Unclonable Function-based mutual authentication scheme, SecAuthUAV, for UAV-GCS. Through comparing with the state-of-the-art authentication protocols [9,10], the authors verified that their proposed scheme could well resist masquerade, replay, node tampering, and cloning attacks over the drone communication channel. On the other hand, many machine learning (ML)-based identification schemes [11,12,13] have also been designed for ensuring drone pilot legal status during the flight procedure. For example, shouFan et al. in [11,12] first verified the pilot’s legal status by monitoring the remote radio controller sending instructions. After analyzing extracted control commands with Linear Discriminant (LD) [14], Quadratic Discriminant (QD) [15], Support Vector Machine (SVM) [16], weighted k Nearest Neighbors (k-NN) [17] and Random Forest (RF) [18] the best performance was achieved with RF approximating to 89% in accuracy. Alkadi et al. in [13] further combined the onboard sensor measurements and received control instructions for improving drone pilot identification performance. Through analyzing with Long Short-Term Memory (LSTM), feature-based, and majority voting-based classification algorithms [19,20,21], the authors concluded that the combination methods could enhance the pilot identification performance. As there exist similarities between automobile driver and drone pilot operations, some algorithms, such as XGBoost [22] and SVM, which have been applied to automobile drivers [23,24], could also achieve impressive performance in drone pilot identification.

**Figure 1:**
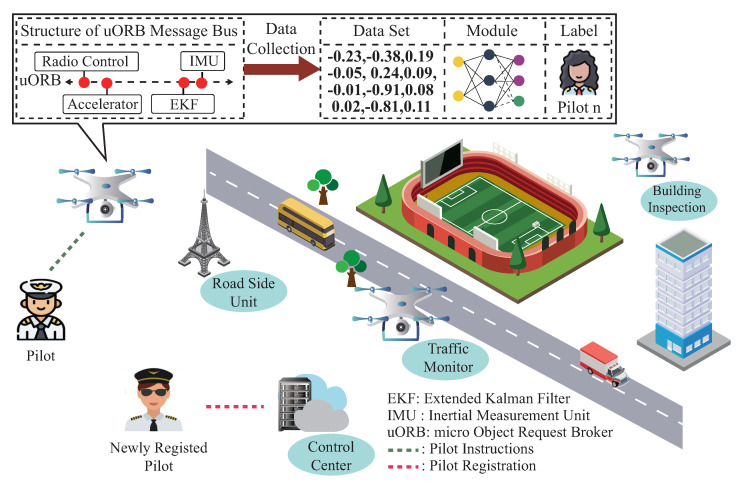
Application scenario.

Despite the significant progress that has been made to reduce drone pilot impersonation attacks, a critical challenge still needs to be addressed: drone pilot membership dynamic management. Unlike the protocol-based authentication scheme, the ML-based identification scheme mentioned in the previous works [11,12,13] could not adapt to newly joined pilots for identification and authentication, only succeeding with the help of the current pilot’s flight data. As illustrated in Figure 1, once the well-established pilot identification scheme is deployed on the flying drone, only the previous registered pilot’s legal status can be verified during the flight procedure. To maintain high identification performance, these ML-based identification schemes require periodic retraining to update their inner parameters. Since the previous registered pilots’ flight data are not accessible due to pilot operation privacy issues, updating ML-based identification schemes would cause the catastrophic forgetting problem, a phenomenon of significant accuracy degradation after training on newly joined pilots’ flight data. Even if the previously registered pilot’s flight data are accessible, storing those data still requires a significant amount of memory. Retraining the ML-based identification scheme from scratch for the newly joined pilots also brings many system overhead costs.

To address the challenge mentioned above, we design a novel task-incremental learning-based drone pilot identification scheme. Motivated by the previous works [12,13,23], we first design a background service to collect drone flight data by subscribing to the topics from a micro object request broker (uORB) message bus. We then construct a module for extracting pilot behavioral traits from the flight data and establish a mapping between the extracted pilots’ behavioral traits and their provided identities. For adapting to the newly joined pilot’s identification, we design an updating mechanism to adjust the inner structure and trainable parameters based on the newly registered pilot flight data. As the proposed scheme possesses high identification accuracy for the newly and previously registered pilots with minimal system overheads, it enhances the potential for protecting drones from pilot impersonation attacks.

The prominent features of this paper are summarized as the following aspects:

- We propose a novel incremental learning-based drone pilot identification scheme for protecting drones from impersonation attacks.
- For obtaining high-quality drone flight data, we design a background service to collect the subscribed topics from uORB message bus without altering the hardware and software architecture.
- To adopt dynamic pilot membership management, we construct an extensible framework and propose an updating mechanism for adopting newly joined pilots into the well-established identification scheme.
- Numerous experiments have demonstrated that the proposed scheme can maintain high identification accuracy for newly and previously registered pilots with minimal system overhead.

The rest of this paper is organized as follows: In Section 2, we first present PX4 inner communication mechanism and adversary model, and then give details about the proposed drone pilot identification scheme and updating mechanism. In Section 3, we conduct systematic experiments to prove the effectiveness of the proposed identification scheme under different environmental settings. We also discuss the advantages and disadvantages of the proposed identification scheme and put forward our future research directions in Section 4. Finally, we provide concluding remarks in Section 5.

## 2. Materials and Methods

### 2.1. UAV Inner Communication Mechanism

With the development of dynamic aerial technology and integrated circuit manufacturing, most flying functionalities have been integrated into flight controllers, such as PX4 [25]. Through utilizing the onboard sensor measurements, PX4 can monitor the drone flight status and provide navigation and mission planning service to the flying drones, reducing the pilot’s operational overhead. According to [25], PX4 has been implemented in Nuttx [26] environment, where different hardware modules have been abstracted into functionalities and provided reliable services for the flight control stack. Moreover, the PX4 also acts as the middleware to intercept the pilot’s instructions and convert them into the preset drone flight attitudes.

Thanks to the inner communication mechanism, the modules within PX4 could cooperate with each other through publishing and subscribing to predefined topics. Specifically, the modules that want to utilize the drone flight attitudes for computation first subscribe to specific topic and then create a listener to receive the onboard measurements at fixed intervals. On the other hand, the modules that want to publish their computation results must also apply for a topic on the uORB message bus and publish their results over the applied topic. Furthermore, PX4 also utilizes the Extended Kalman Filter [27] algorithm to fuse the onboard sensor measurements and publish high-precision drone flight attitude over the uORB message bus, reducing the side effects caused by external environmental conditions. Since the high-precision flight data run over the uORB message bus, we design a background service to subscribe to the predefined topics listed in Table 1 from the uORB message bus and utilize them for drone pilot identification.

### 2.2. UAV Pilot Impersonation Attack

**Figure 2:**
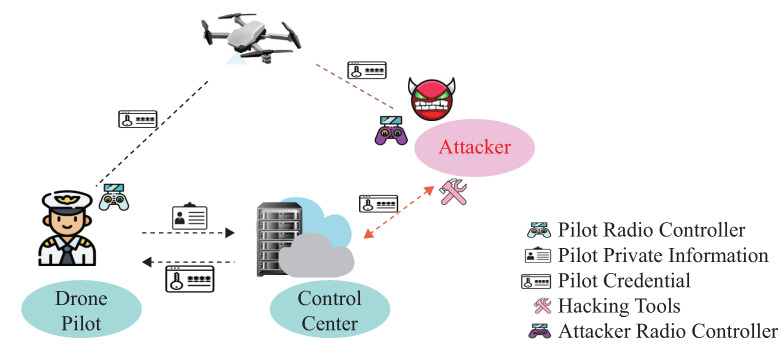
Impersonation attack.

As drones deployed in the open environment carry sensitive information, many adversaries try to capture flying drones for malicious purposes. Compared with other electronic attacks, such as GPS spoofing attacks or Man-in-the-middle attacks, the adversary could launch an impersonation attack by utilizing the compromised credential, as illustrated in Figure 2. This paper assumes that all pilots and flying drones must first complete registration at the ground control center. After that, the pilot can utilize their credentials for mutual authentication at the beginning of the communication. Once their legal status is verified on the drone side, the pilot uses their remote radio controller to send instructions for the flying drone. On the other hand, the adversary tries to utilize hacking tools to capture the pilot’s credentials from the ground control center. Once the victim pilot’s credentials are obtained, it utilizes the compromised credentials to obtain the flying drone’s control privilege. As the knowledge-based authentication scheme can not verify the pilot’s legal status during the flight procedure, the adversary can impersonate the victim pilot and utilize their radio controller to send malicious instructions to the flying drone.

To protect drones from impersonation attacks, we propose a pilot identification scheme that verifies the pilot’s legal status during the flight procedure. As distinct differences exist between the victim pilot and adversary operation profile, the proposed identification scheme will differentiate the adversary flight data from the current pilot’s. If the estimated identity does not match the pilot-provided credential, an alert will be triggered by this identification system. At the same time, the PX4 flight controller will stop executing the received instructions and switch to automatic mode, guiding the drone to land at the take-off place to stop further impersonation attacks.

### 2.3. Incremental Learning

Incremental learning was first proposed by Schlimmer in [28], whose goal is to enable machine-learning models to continuously adjust their structure according to the newly generated data, adapting to the dynamic changing environment. In recent years, incremental learning has become an important research topic and is applied to many application scenarios, such as wireless device identification, smartphone counterfeit detection, and natural language processing [29,30,31]. In order to adapt to the dynamic changing environment, many researchers have proposed to utilize regularization approaches to mitigate the catastrophic forgetting problem. For example, Kirkpatrick et al. in [29] proposed an elastic weight consolidation (ewc) approach to calculate a diagonal of the Fisher Information Matrix. They assumed that the model would learn the importance after each task while ignoring the influence of those parameters along the learning trajectory in weight space. To address the importance overestimating problem, the authors in [30] proposed memory aware synapses (mas) to fuse Fisher Information Matrix approximation and online path integral in a single algorithm to calculate the importance for each parameter. Furthermore, the authors in [32] proposed an incremental learning method, learning without forgetting (lwf), to regularize data drift with the temperature-scaled logits during the training procedure. Instead of the previously mentioned works [29,30,32], there also exist some techniques, such as rehearsal approaches [32,33,34], that have been proved to be effective in improving incremental learning performance. To our knowledge, the proposed identification scheme is the first work that utilizes incremental learning for drone pilot identification. We compare the performance of our updating mechanism with the state-of-the-art incremental learning algorithms, such as [29,30,32] to illustrate the effectiveness of the proposed algorithm. Systemic experiments in natural and constrained enviroments have demonstrated that the proposed updating strategy has higher identification accuracy for the previously and currently registered pilots over the compared algorithms.

### 2.4. Problem Definition

We define the learning on newly joint pilot drone flight data as a new task in our drone pilot identification scheme. When training on *t*-th pilot’s drone flight dataset $D_{t}$, we assume no direct access to ${\left\{D_{k}\right\}}_{k=1}^{t-1}$ for the moment, leading to the following training objective.

(1) $$L\left(D_{t};w\right)=\frac{1}{\left|D_{t}\right|}\sum_{\left(x,q\right)\in D_{t}}\ell \left(g_{w}\left(x\right),q\right),$$

where $x\in R^{N}$ and q∈R denote the *N*-dimensional drone flight data and the corresponding pilot-provided identity, respectively. $g_{w}\left(x\right)$ represents the well-established pilot identification model parameterized by a vector *w*. *ℓ* is the objective function, quantifying drone pilot identification performance. One may add a regularizer r(w) to Equation (1) to gain resistance to catastrophic forgetting. For evaluation, we may measure the performance of $g_{w}\left(x\right)$ on the hold-out test sets of all tasks seen so far.

(2) $$\sum_{k=1}^{t}L\left(V_{k};w\right)=\sum_{k=1}^{t}\left(\frac{1}{\left|V_{k}\right|}\sum_{\left(x,q\right)\in V_{k}}\ell \left(g_{w}\left(x\right),q\right)\right),$$

where $V_{k}$ is the test set for the *k*-th task. An ideal drone pilot identification scheme should identify well on all newly joined pilots and endeavor to mitigate catastrophic forgetting of the previous pilots, resulting in better identification performance.

### 2.5. Pilot Identification Based on UAV Flight Data

#### 2.5.1. Data Collection and Preprocessing

In order to collect highly precise drone flight data and minimize the side impact caused by the external environment, we have designed a background service to subscribe to the selected topics from the uORB message bus instead of the ground control center. Due to the unstable connections caused by external weather conditions and drone mobility, the integrity of data transmitted to the ground control center over the mavlink protocol has been broken, downgrading drone pilot identification performance. According to the PX4 documentation, there are a total of 54 topics running through the uORB message bus, designed explicitly for the quadcopter framework. In order to select the most representative attributes which can describe the pilot behavioral traits, we utilize the embedded feature selection algorithm [35] to filter out the irrelevant topics and attributes. Specifically, we first select five pilots’ flight data to construct a mini dataset, which includes drone flight attitude, inner communication message, and pilot-provided identity. We then utilize RF as a classifier to identify drone pilot identity based on the attribute within subscribed uORB topics. Finally, we sort the attributes by identification accuracy in descending order and preserve the first 28 attributes for constructing the identification scheme. Table 1 provides details about the selected topics and preserved attributes.

As illustrated in Table 1, the selected topics are mainly about the received pilot instructions, drone flight attitudes, and the inner control commands sent from the flight controller to the executor. Due to internal hardware errors and external factors, significant deviations exist among the selected attributes. To address this problem, we first utilized the Kalman filter algorithm to find the abnormal deviations from the selected topics and then replaced this deviation with the previous observation. Note that the selected topics maintain their publishing frequency, and the preserved attributes have their working dimensions. We implemented a sliding window with one-second width to streamline the selected attributes to 1 Hz, where the average value within this window is utilized for current observation. We then applied the following standard equation to normalize the observations before feeding them into the drone pilot identification scheme.

(3) $$x^{\prime }=\frac{x-x_{mean}}{x_{std}},$$

where *x* represents the previous generated observations, $x_{mean}$ and $x_{std}$ are mean and standard deviation of current observations. After data processing, we concatenated the selected attributes chronologically and generated the input sequence with 28 dimensions. Each time, we selected 64 consecutive observations to form one input sequence map, and the overlap between consecutive maps is kept at 32. Furthermore, we digitized pilot-provided credentials as unique numbers and utilized these numbers to represent the ground truth while constructing the identification scheme.

#### 2.5.2. Drone Pilot Identification

**Figure 3:**
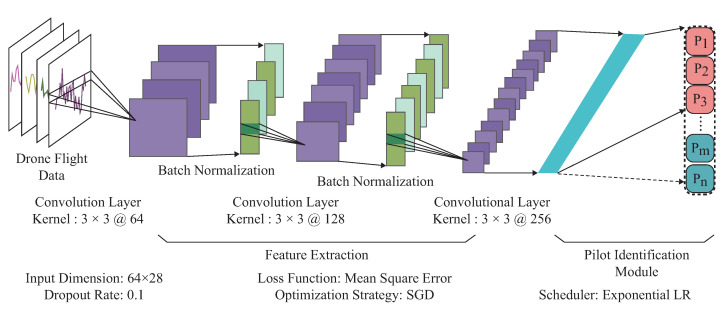
Incremental learning-based UAV pilot identification.

In order to make the drone pilot identification scheme adaptable for dynamic membership management, we first designed a foundation framework that could estimate pilot identity according to the drone flight data. Motivated by the previous work [36,37], this foundation framework consists of feature extraction and pilot identification module, as illustrated in Figure 3. In the feature extraction module, we concatenated three convolution layers sequentially and appended the batch normalization operation at the output of each convolution layer for the pilot behavioral traits extraction. The convolutional layer utilizes the local parameter-sharing mechanism for extracting the spatial and temporal relationships among the input drone flight data. Furthermore, batch normalization has been applied to recenter and rescale the output features of the convolutional layer, boosting the gradient backpropagation and reducing the probabilities of the gradient disappearance problem. As the negative representations extracted by each convolutional layer have physical meaning when describing drone flight data, we eliminated the ReLU [38] functions in the constructed foundation framework.

In the pilot identification module, we utilized one full connection layer to establish connections between the extracted hidden representations with the registered pilot identity. The full connection layer could assign appropriate weights for each extracted hidden representation, just like an affine matrix. By utilizing the backpropagation mechanism, the full connection layer can approximate the pilot’s real identity by adjusting the trainable weights. In order to make this identification scheme adaptable for dynamic pilot membership management, the form of the full connection layer also can be altered according to the number of currently registered pilots. As illustrated in Figure 3, we first utilized the drone flight data as input to the pilot identification scheme. Through a series of convolutional operation and the batch normalization, we extract pilot behavioral traits step by step, where the purple blocks represent the convolutional features and green blocks indicate the batch normalization features. After that, we flatten the hidden representations into one dimension(cyan blocks) and establish the connections between the hidden representations with predicted pilot identity, where the red blocks represent the previously registered pilots, and the green ones indicate the newly joined pilots. Once newly registered pilots have been integrated into the drone system, the green blocks will be appended at the end of the red ones in the previously constructed identification scheme. Regarding the identification scheme updating mechanism, we utilized the previously constructed identification scheme to guide the newly generated identification scheme only with the help of newly registered drone pilot flight data. For more details about updating mechanism, please refer to Section 2.5.3.

Regarding the optimization strategy, we utilized the least mean square error to calculate the distance between the predicted pilot identity and the ground truth. The loss function can be expressed as Equation (4).

(4) $$loss_{mse}=\frac{1}{n}\sum_{i=1}^{n}\left|P_{i}-P_{r}\right|,$$

where *n* is the number of identities the proposed identification scheme could handle each time. $P_{i}$ is the proposed identification scheme predicted identity, and $P_{r}$ is the ground truth corresponding to the pilot-provided credential. |·| represents the hamming distance, where zero denotes that the estimated identity is consistent with the generated ground truth. One represents that the estimated identity deviates from the generated ground truth. Figure 3 illustrates the hyperparameters we used to construct the pilot identification scheme, and we applied stochastic gradient descent optimization [39] strategy to find the parameters for best identification performance.

#### 2.5.3. Drone Pilot Identification Updating Mechanism

Once the pilot completes registration at the ground control center, he will be granted the credential for obtaining the drone control privilege. In order to validate this pilot’s legal status during the flight procedure, we first required the pilots to utilize a remote radio controller to send instructions to the drone for completing some basic flight missions. Meanwhile, we utilized the previously designed background services to extract the flight data $X_{n}$. We digitized their credential into a unique number to represent their identity in our identification scheme. Since the other registered pilot’s flight data are inaccessible due to their operation privacy, we only utilized the $X_{n}$ and the previous well-constructed identification scheme to optimize parameters of the altered network structure, aiming at high identification accuracy for all registered pilots.

As illustrated in Figure 3, we first parameterized this identification scheme with parameter $\theta_{s}$ and $\theta_{o}$, where $\theta_{s}$ represents the parameters within feature extraction module and $\theta_{o}$ indicates the parameters within pilot identification module. When updating the pilot identification module for newly registered pilots, we first added nodes to the output layer (the green blocks in Figure 3). After that, we established the connections $\theta_{n}$ between the feature extraction module and the newly added nodes. The number of connections equals the number of newly added pilots times the number of the output features extracted by the feature extraction module. We initialized $\theta_{n}$ with random Gaussian distribution and then updated this identification scheme in the following procedure. At the beginning of updating procedure, we first froze parameter $\theta_{s}$ and $\theta_{o}$ and utilized newly registered pilot flight data $X_{n}$ to training parameter $\theta_{n}$ until to coverage. We then utilized $X_{n}$ to train all the network parameters including $\theta_{s}$, $\theta_{o}$ and $\theta_{n}$ to converge. As we could only utilize the currently registered pilot data $X_{n}$ for updating the drone pilot identification scheme, the optimization target for newly registered pilots is to minimize the distance between the predicted identity and pilot-provided ground truth. We utilized the Equation (5) to describe the newly registered pilot’s optimization target.

(5) $$L_{new}\left(y_{n},{\hat{y}}_{n}\right)=-y_{n}\cdot log{\hat{y}}_{n},$$

where $y_{n}$ is the output of the pilot identification scheme based on pilot-provided data $X_{n}$ and ${\hat{y}}_{n}$ is the corresponding ground truth. As for the previously registered pilot optimization, we utilized the Knowledge Distillation loss [40] to encourage the current pilot identification scheme’s output to approximate the previous one’s outputs.

(6) $$L_{old}\left(y_{o},{\hat{y}}_{o}\right)=-\sum_{i=1}^{l}{y^{\prime }}_{o}^{\left(i\right)}log{\hat{y^{\prime }}}_{o}^{\left(i\right)},$$

where *l* is the number of the previously registered pilots in each iteration, ${y^{\prime }}_{o}^{\left(i\right)}$ indicates the probabilities predicted by the previous well-constructed identification scheme, and ${\hat{y^{\prime }}}_{o}^{\left(i\right)}$ is the probabilities estimated by the newly constructed identification scheme. Furthermore, we tried to regularize the parameters $\theta_{s}$, $\theta_{o}$ and $\theta_{n}$ with R with the decay of 0.005 to force the newly constructed identification focus on the newly registered pilot behavioral traits.

In order to minimize the distance between the current and previous identification scheme output probabilities, we utilized the Equation (7) to aggravate the small probabilities.

(7) $$y_{o}^{\prime (i)}=\frac{{\left(y_{o}^{\left(i\right)}\right)}^{1/T}}{\sum_{j}{\left(y_{o}^{\left(j\right)}\right)}^{1/T}},\;{\hat{y}}_{o}^{\prime (i)}=\frac{{\left({\hat{y}}_{o}^{\left(i\right)}\right)}^{1/T}}{\sum_{j}{\left({\hat{y}}_{o}^{\left(j\right)}\right)}^{1/T}}$$

where we utilized hyper-parameter *T* to control the scale factors to amplify the small probabilities for each predicted pilot identity. Since drone pilot identification is a multi-label classification problem, we took the sum of loss for old and new tasks in each iteration. Algorithm 1 gives more details about updating procedure proposed in the drone pilot identification scheme. In order to further improve identification performance, we preserved 100 samples for each previously registered pilot flight data. We merged the newly registered pilot flight data for this newly generated identification scheme. We used Pilot Identification to indicate the previous well-constructed identification scheme, and the $\lambda_{o}$ controls weights between the old and current tasks. After conducting system experiments, we find that the parameter $\lambda_{o}$ with 0.1 could achieve the best identification performance for all registered pilots.
**Algorithm 1:** Drone Pilot Identification Updating Algorithm
1: **Start:**
  $\theta_{S}:$ feature extraction parameters
  $\theta_{o}:$ identification parameters for the previous registered pilots
  $\theta_{n}:$ added parameters for newly registered pilots
  $X_{n},Y_{n}:$ newly registered pilot’s drone flight data and their identity 
2: **Initialize:**
  $Y_{o}\leftarrow$ Pilot Identification$\left(X_{n},\theta_{s},\theta_{o}\right)$
  $V_{n}\leftarrow$ RANDINT $\left(|Y_{n}|\right)$
3: **Train:**
  Define ${\hat{Y}}_{o}$ = Pilot Identification($X_{n},\theta_{s},\theta_{o}$) ▹ previous registered pilot
  identity estimation
  Define ${\hat{Y}}_{n}$ = Pilot Identification($X_{n},\theta_{s},\theta_{n}$) ▹ newly registered pilot
  identity estimation
  $\theta_{s}^{*}$, $\theta_{o}^{*}$, $\theta_{n}^{*}$←$\underset{\theta_{s},\theta_{o},\theta_{n}}{\arg\min}$ ($\lambda_{o}L_{old}\left(Y_{o},{\hat{Y}}_{o}\right)+L_{new}\left(Y_{n},{\hat{Y}}_{n}\right)+R\left(\theta_{s},\theta_{o},\theta_{n}\right)$)


## 3. Results

### 3.1. Environmental Setting

**Figure 4:**
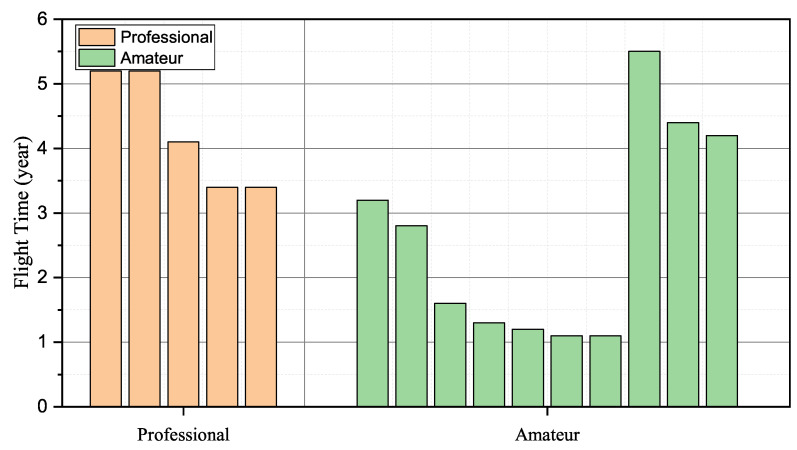
Pilot operation proficiency.

As the quadcopter has received wide attention due to its easy operation and broad application, we conducted experiments on the quadcopter, such as P450 and S500, to validate the effectiveness of the proposed identification scheme. In data collection, 15 students were required to fly quadcopters in the natural and constrained environment. Regarding P450, produced by AmovLab, it was installed with a depth of field and optical flow sensors to provide reliable flight performance for the drone. When it comes to S500, we constructed it from scratch under the guidance of the CUAV flight stack. We utilized the remote radio controller Futuba to capture the pilot instructions and send control signals to the flying drone. Among the invited pilots, five students from AeroModelling Team were required to act as professional pilots, and the remainder who came from our laboratory were the amateurs. Figure 4 details the participants’ operation proficiency, where we utilized the flying times to represent their operation experience.

**Figure 5:**
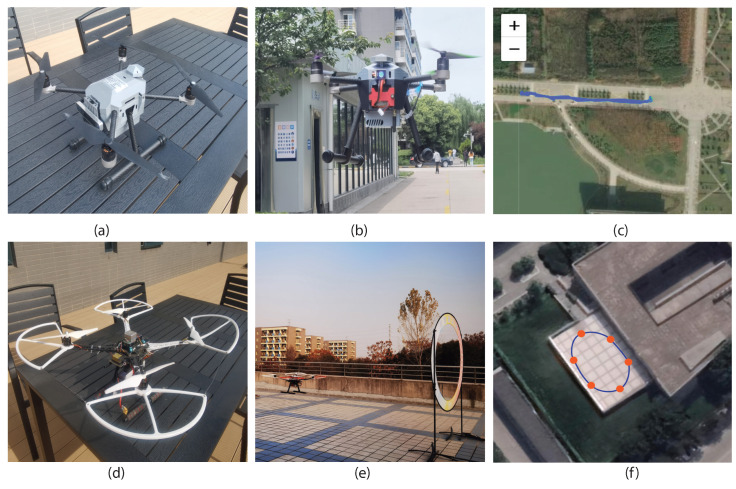
Experimental settings. All the experiments have been conducted on drone P450 (**a**) and S500 (**d**). We have invited all the pilots to fly the drone in natural (**b**) and constrained (**e**) environments. The flight trajectories are illustrated in (**c**,**f**).

To validate the effectiveness of the proposed identification scheme, we have conducted experiments over P450 and S500, as illustrated in Figure 5. We first required participants to fly P450 in the natural environment for a traffic monitoring mission, the most common flight task in the quadcopter. Specifically, the pilots utilized a remote radio controller to send instructions to the drone for taking off, hovering in the air, and landing at the predefined destination. At the same time, the pilots were asked to record three-minute videos about the traffic conditions. Furthermore, we conducted experiments in the constrained environment, whose settings complied with [12,13]. We had preset the flight trajectories and asked pilots to control drones to pass through the way-points without collision. To reduce the side effects caused by external weather conditions, all the data were collected in the constrained environment on sunny days.

We also conducted experiments on S500 in natural and constrained environments with similar settings to further illustrate the effectiveness of the proposed identification scheme. To collect sufficient data, all the participants were required to fly the drone ten times, and we utilized the designed background service to monitor the drone flight status. Regarding the ground truth generation, we digitized the pilot-provided credential and mapped it as a unique number, indicating the ground truth in our experiments. Table 2 gives more details about the experimental settings and the portion of the collected dataset has been available on 15 June 2023 at https://github.com/FRTeam2017/DronePilotIdentification.git.

### 3.2. The Hardware and Software Architecture

In order to collect high-precise drone flight data, we first designed a background service to subscribe to the selected topics from the uORB bus. In data preprocessing, Anaconda is utilized to create a pure Python environment, where the package Pyulog is installed for converting the collected flight data into CSV format, and Pandas is used for calculating statical features. As for the drone pilot identification scheme implementation, we utilized the Pytorch framework to extract pilot behavioral traits from the flight data and estimate their identity in real-time.

In the hardware configuration, we have constructed a workstation equipped with an i7-8700 CPU processor and 24 GB of memory. In order to accelerate the training procedure, an NVIDIA-3080 graphics card was installed for parallel computing. We utilized Ubuntu 20.04 to manage the previously mentioned hardware equipment, and the above configuration determines the results presented below.

### 3.3. Drone Pilot Identification Based on P450

In this section, 15 pilots were required to utilize P450 for completing flight missions in the natural and constrained environment, as illustrated in Figure 5. In order to collect sufficient drone flight data, we asked them to repeat the flight mission ten times in the natural and constrained environment separately. We utilized the first eight trajectories for training and the remainder for testing that partition obeys the machine learning and pattern recognition algorithm. Furthermore, there is no overlapping between the training and testing trajectories. After data preprocessing, we obtained 54,123 training and 11,254 testing samples in the natural environment. We also obtained 49,938 training and 11,432 testing samples in the constrained environment. Table 3 details drone pilot identification performance in different environmental settings based on P450, where P* represents the sample that drone pilot identification scheme will be classified, and T* indicates the estimation results based on given flight data.

This table demonstrates that the proposed identification scheme can achieve impressive performance based on the selected topics on uORB message bus. In the natural environment, the average identification accuracy approximates 94.87%, and the average identification accuracy for the constrained environment is about 95.71%, slightly better than the natural environment. One possible explanation is that external weather conditions, such as wind and magnets, may affect the pilot’s operation behaviors, decreasing identification performance. Note that the worst identification accuracy is 73.54%. The proposed identification scheme can be deployed on the drone for real-time pilot identification.

### 3.4. Drone Pilot Identification Based on S500

In order to further illustrate the effectiveness of the proposed identification scheme, we also conducted the same experiments in S500. We required all the pilots to utilize S500 for traffic monitoring in the natural environment and pass through the arch door preset along the waypoints in the constrained environment. To collect sufficient drone flight data, all the pilots were asked to repeat the mission ten times. We utilized the flight data from the first eight times for training and the last two for testing. After data preprocessing, we obtained 62,118 flight samples in the natural environment and 58,921 in the constrained environment. Table 4 provides details about the performance of the proposed drone pilot identification, and the meaning of P* and T* has been illustrated in Table 3.

The proposed drone pilot identification scheme achieves similar accuracy on drone S500. According to Table 4, eight pilot identification accuracy exceeds 95% The minimal identification accuracy has been achieved with 82.17% (constrained environment) and 88.47% (natural environment). The reason of the minimal identification accuracy is also caused by the external weather condition factors. As the average identification accuracy on S500 is 93.95% and 94.23% in natural and constrained environments, respectively, we can conclude that the proposed scheme can maintain high identification performance over different quadcopters.

### 3.5. Performance Comparison

Since there is no public drone pilot identification dataset available and this is the first work that utilizes the incremental learning paradigm for drone pilot identification, we first compared the proposed identification performance with the most related works [11,12,13] in terms of objectiveness, the number of participants, and utilized signals et al. in Section 3.5.1. We then compare the identification performance with the algorithms mentioned in the related works in Section 3.5.2. Finally, we compare our updating mechanism with the off-the-shelf incremental algorithms [29,30,32] to illustrate effectiveness of the proposed identification scheme in Section 3.5.3.

#### 3.5.1. Comparison with the Related Works

In this section, we first compare the proposed identification scheme with the most related works [11,12,13] in terms of the experimental setting, objectiveness, number of participants, utilized signals, and identification performance. Table 5 provides details about the comparison results. As the compared works do not provide the source code and drone flight data, we utilized the reported results for the comparison.

As illustrated in Table 5, we employed similar experimental settings to demonstrate the effectiveness of the proposed identification scheme. For example, we used the quadcopter to collect drone flight data and verify the pilot’s legal status. Compared with the related works, we verified the effectiveness of the proposed identification scheme in natural and constrained environments. We also have to consider the extensibility when constructing drone pilot identification scheme in our work. Although the identification accuracy is not comparable due to lacking the public dataset, the proposed scheme achieves the best identification accuracy with 95.24% over 15 pilots, approximating to the best results reported in the related work [13].

#### 3.5.2. Comparison with the Algorithms Mentioned in Related Works

We also compared our identification scheme with the algorithms mentioned in the related works [11,12,13,23]. Specifically, we compared the algorithm QD, LD (solver = SGD), Bagging, RF ($n_{e}stimator$ = 100), Adaboost, DT mentioned in work [12], and LSTM ($num_{l}ayer$ = 2), Feature (estimator = RF, n_estimator = 5), Voted (base_estimator=SVC, n_estimator = 5) mentioned in [13]. Furthermore, the algorithms, including SVM, XGB, and RF mentioned in [23] are utilized for comparison due to their competitive performance. Due to the lack of a public dataset, all the comparison algorithms were tested on our collected dataset. The best parameters for the compared algorithms are set with the grid search algorithm [41]. In order to reduce side effects caused by data preprocessing, all the data preprocessing procedures align with the original works. Table 6 provides more details about the drone pilot identification performance.

Table 6 and Table 7 give details about the identification performance of the proposed identification scheme and the related works with over 15 participants. Our proposed identification scheme achieves the best identification performance over 15 participants compared to the related algorithm. Specifically, four out of fifteen pilots’ identification are 100%, and the fifteenth pilot with 95.42% has achieved minimal identification accuracy. One reason is that the identification scheme can effectively use the spatial and temporal relationships between the subscribed UAV flight data.

#### 3.5.3. Comparison with the SOTA Incremental Learning Algorithms

**Figure 6:**
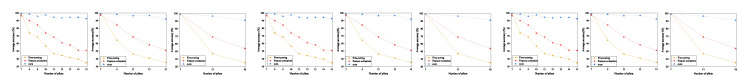
Performance comparison versus different incremental step.

In order to validate the effectiveness of the proposed updating mechanism, we conducted experiments over the quad-copter S500 flight data. We first compared the proposed updating mechanism with the feature extraction and fine-tuning mentioned in the related work [42,43], where the feature extraction tries to fix the shared parameters $\theta_{s}$ and $\theta_{o}$ and utilize the newly joined pilot light data as a new task for training $\theta_{n}$, While the Fine-tuning utilized $\theta_{s}$ and $\theta_{n}$ for learning a new task and maintain the previous task-specific parameters $\theta_{o}$ stable. Each time we utilized the fixed number of newly joined pilot flight data to update the proposed identification scheme and the average accuracy up to the currently registered pilots (mentioned in Equation (2)) to evaluate the drone pilot identification performance. From Figure 6, we can see that the proposed could maintain high identification performance with the increased size of the registered pilots in different incremental steps. However, Fine-tuning and Feature extraction suffer from the catastrophic forgetting problem. Note that the performance of feature extraction is relatively better than fine-tuning. One reason to explain this is that the feature extraction updating mechanism fixes the shared parameters $\theta_{s}$ and $\theta_{o}$, which keeps more historical information during the updating procedure.

We also compared the proposed updating mechanism with the state-of-the-art incremental learning algorithms, including lwf [32], ewc [29] and mas [30]. These methods have been utilized as the standard comparison algorithms to illustrate the effectiveness of incremental learning-based identification framework in the IoT device recognition and identification [44,45]. As illustrated in Table 8, we first selected seven pilot’s flight data to initialize the proposed identification scheme. After that, we utilized the different number of pilot flight data to update the well-established identification scheme and the up-to-current registered pilot average accuracy to depict performance among the compared algorithms. It is clear that the well-established drone pilot identification performance decreases with the increased number of newly joined pilots. Take mas for example. The identification performance of mas dropped from 98.11% with seven registered pilots to 61.52% with 15 registered pilots. One of the reasons to explain this is that the mas needs many current pilot flight data to determine the importance of each shared parameter, which contradicts the setting of drone pilot identification. Compared with the other SOTA incremental learning algorithm, our proposed updating mechanism could maintain high identification performance with the increased number of registered pilots. Based on the previous analyzes, the updating mechanism can help the drone pilot identification scheme adopted for newly registered pilots in dynamic pilot membership management.

### 3.6. Time and Space Complexity

As a drone is a lightweight system, the time and space overhead is critical for timely protection of the drone from impersonation attacks. According to Figure 3, the proposed identification scheme consists of pilot behavioral trait extraction and a pilot identification module. The pilot behavioral extraction module comprises convolution layers and batch normalization operations. Based on [46], the time complexity for each convolution layer is $O(M^{2}\times K^{2}\times C_{in}\times C_{out})$, where *M* is the dimension of the input feature map, *K* is the size of the convolutional kernel, $C_{in}$ and $C_{out}$ are the number of input and output channels. As for batch normalization, its time complexity depends only on the input feature maps, O(1). We used the full connection layer to map the hidden representation to the pilot identity for pilot identification. Thus, the time complexity for the full connection layer is the same as batch normalization. The proposed identification scheme involves extracting behavioral traits and estimating identity in a sequential manner. The depth of the identification module determines the time complexity of this process. It is represented by the equation: $O(\sum_{l=1}^{D}M^{2}\times K^{2}\times C_{in}\times C_{out})$, where *D* is the depth of the proposed identification module. As for run time overhead, the Python built-in time function suggests that the proposed scheme only needs 0.031 s for drone pilot identification each time. Furthermore, we implemented a Pytorch-implemented parameter statistical function to assess the system overhead. Our identification scheme only has 13 M parameters, indicating that it requires reasonable storage overhead for identification.

## 4. Discussion

In this paper, we propose a novel drone pilot identification scheme for protecting drones against impersonation attacks and an updating mechanism for adopting to dynamic pilot membership management. In order to validate the effectiveness of the proposed identification scheme, we have conducted experiments over P450 and S500 in different environmental settings. Despite the impressive results that have been produced, there are still existing challenges in our proposed identification scheme.

First, the identification performance could also be further improved. The numerical results in Table 3 and Table 4 have shown that the proposed identification scheme could identify most pilots with high identification accuracy. However, some pilots, such as the tenth pilot in Table 3 still could not be well identified. One explainable reason is that external weather conditions, such as wind, could change the pilot’s behavioral traits for maintaining stabilization during the flight procedure, and the proposed identification scheme does not consider the pilot’s behavioral traits in different weather conditions. In our future work, we will design a more intelligent identification scheme that can utilize weather condition robust features for drone pilot identification.

Second, different application scenarios should be utilized to verify the proposed updating mechanism further. This paper validates the proposed updating mechanism for adopting newly joined pilots under similar experimental settings. In the application scenario, the newly joined pilots’ flight data could come from different environments, which may bring side effects on the performance of the proposed identification scheme. Furthermore, the registered pilots’ leaving scenario should also be considered to enhance dynamic drone pilot membership management.

Third, more experiments should be conducted on different types of drones to validate the effectiveness of the proposed identification scheme. This paper only verified the effectiveness of the proposed identification scheme on P450 and S500 in the preset natural and constrained environment. As more types of drones have been designed and devoted to application scenarios, a more general and robust identification scheme must be deployed on the drone side to further reduce pilot impersonation attacks.

With an increasing number of drones being deployed to real application scenarios, pilot legal status verification will become a dispensable part of the drone system. In the near future, we will conduct more research about drone related attacks and design a more lightweight and robust pilot identification to enhance drone flight security.

## 5. Conclusions

This paper has presented a novel task incremental learning-based drone pilot identification scheme to protect drones from pilot impersonation attacks and adopt to dynamic pilot membership management. In order to verify the effectiveness of the proposed identification scheme, we conduct systemic experiments on P450 and S500 in different environmental settings. The numerical results show that the proposed identification scheme achieves the best identification accuracy with 95.71% on P450 and 94.32% on S500 over 15 pilots, respectively. Furthermore, the proposed scheme only consumes 13M parameters and can complete drone pilot identification within 0.031 s. Due to the high identification accuracy and low system overhead, the proposed drone pilot identification scheme demonstrates great potential to protect drones from impersonation attacks. In the future, we will consider more factors for designing an intelligent drone pilot identification scheme and conduct comprehensive experiments using different types of drones and environmental settings to verify the robustness of the proposed identification scheme. 

## Figures and Tables

**Table 1:** Selected Topics and Its Physical Meaning.

Topic	Attribute	Minimize	Maximize	Frequency	Description
received instructions	values[0:4]	1200	1800	2 Hz	pilot
					instructions
executor control	controls[0:4]	0.5	1.5	20 Hz	flight controller
					instructions
actuator output	output[0:4]	900	2100	10 Hz	instructions for
					ESC
accelerator	x, y, z	−30	30	1 Hz	acceleration in
					body frame
gyroscope	x, y, z	−5	5	1 Hz	angular velocity
					in body frame
magnetometer	x, y, z	−2	2	1 Hz	magnet in
					NED
angular velocity	xyz[0:3]	−5	5	100 Hz	angular velocity
					in NED
local attitude	q[0:4]	−1	1	10 Hz	flight attitude
					in quaternions

**Table 2:** Environmental settings.

	UAV	Environment	Participates	Routines	Samples
1	P450	Nature	15	150	65,337
		Constrained			61,370
2	S500	Nature	15	150	62,118
		Constrained			58,921

**Table 3:** Drone pilot identification in natural and constrained environment based on P450.

	T1	T2	T3	T4	T5	T6	T7	T8	T9	T10	T11	T12	T13	T14	T15
**P1**	100.0	0.	0.	0.	0.	0.	0.	0.	0.	0.	0.	0.	0.	0.	0.
	97.73	0.	0.	0.81	0.	0.28	0.	1.11	0.	0.	0.	0.	0.	0.	0.
**P2**	0.	99.12	0.	0.	0.	0.	0.	0.	0.	0.	0.81	0.	0.	0.	0.
	0.	99.43	0.	0.	0.	0.56	0.	0.	0.	0.	0.	0.	0.	0.	0.
**P3**	0.	0.	88.78	0.	0.	0.	0.	0.	11.23	0.	0.	0.	0.	0.	0.
	0.	0.	98.91	1.12	0.	0.	0.	0.	0.	0.	0.	0.	0.	0.	0.
**P4**	0.	0.	0.	95.54	0.	0.	0.	0.	0.	0.	3.98	0.54	0.	0.	0.
	0.	0.21	0.59	99.22	0.	0.	0.	0.	0.	0.	0.	0.	0.	0.	0.
**P5**	0.	0.	0.84	0.	85.12	9.71	0.	0.	0.	0.	0.	1.22	0.	0.	3.01
	0.	0.	0.	0.	97.36	0.	2.67	0.	0.	0.	0.	0.	0.	0.	0.
**P6**	0.	0.	0.	0.	0.	99.24	0.76	0.	0.	0.	0.	0.	0.	0.	0.
	0.	0.	2.56	0.	0.	96.23	0.	0.	1.23	0.	0.	0.	0.	0.	0.
**P7**	0.	0.	0.	0.	0.	8.7	91.22	0.	0.	0.	0.	0.	0.	0.	0.
	0.	0.	0.	0.	0.	0.	98.22	0.	0.	1.75	0.	0.	0.	0.	0.
**P8**	0.	0.74	0.	0.	0.	0.	0.	99.2	0.	0.	0.	0.	0.	0.	0.
	0.	0.	0.	0.	0.	0.	0.	100.	0.	0.	0.	0.	0.	0.	0.
**P9**	0.	0.	3.92	0.	0.	2.09	0.	0.	89.72	0.	0.	0.	0.	0.	4.13
	0.	0.	0.	0.	0.	0.	0.	0.	100.	0.	0.	0.	0.	0.	0.
**P10**	0.	0.	0.	21.72	0.	0.	0.	0.	0.	73.54	3.54	0.	0.	1.08	0.
	0.	0.	0.	0.	0.46	0.	0.	0.	0.	98.32	0.	0.	1.22	0.	0.
**P11**	0.	0.	0.	0.65	0.	0.	0.	0.	0.	1.08	87.82	9.78	0.	0.	0.65
	0.	0.	0.	0.	0.	0.	0.	0.	3.09	0.71	96.22	0.	0.	0.	0.
**P12**	0.	0.	0.	3.78	0.	0.	0.	0.	0.	0.	7.14	89.12	0.	0.	0.
	0.	0.	0.	0.	0.	0.	0.	0.	0.	0.	0.	100.	0.	0.	0.
**P13**	0.	0.	0.	0.	0.	0.	0.	0.	0.	0.	0.	0.	100.	0.	0.
	0.	0.	0.	0.	0.	0.	0.	0.	0.	0.	0.	0.	100.	0.	0.
**P14**	0.	0.	0.	0.	0.	0.	0.	0.	0.	0.	0.	0.	0.	100.	0.
	0.13	0.	0.	0.	0.	0.	0.	0.	0.	0.	0.	0.14	0.	99.36	0.43
**P15**	0.	0.	0.	0.	0.	2.26	0.	0.	0.	0.	0.	2.95	0.	0.	94.72
	0.	0.	0.	0.	0.	1.45	0.	0.	0.	0.	0.	0.62	0.	0.	97.92

**Table 4:** Drone pilot identification in natural and constrained environment based on S500.

	T1	T2	T3	T4	T5	T6	T7	T8	T9	T10	T11	T12	T13	T14	T15
**P1**	100.0	0.	0.	0.	0.	0.	0.	0.	0.	0.	0.	0.	0.	0.	0.
	94.56	0.	0.	0.	0.	5.44	0.	0.	0.	0.	0.	0.	0.	0.	0.
**P2**	0.	91.54	0.	0.	0.	0.12	0.	0.	0.	0.24	0.	6.67	0.	0.	1.43
	0.	96.07	0.95	0.	0.	0.	0.	0.	0.	0.	0.	2.26	0.	0.	0.72
**P3**	0.	10.01	88.49	0.	0.	0.	0.	0.	0.	0.15	0.	0.	0.	0.	1.35
	0.	7.74	91.48	0.	0.	0.	0.	0.	0.	1.05	0.	0.	0.	0.	0.
**P4**	0.	0.	0.	100.	0.	0.	0.	0.	0.	0.	0.	0.	0.	0.	0.
	0.	0.	0.	100.	0.	0.	0.	0.	0.	0.	0.	0.	0.	0.	0.
**P5**	0.42	0.	0.	0.	91.98	0.	0.42	0.	0.	0.	0.	0.	0.	7.17	0.
	0.	0.	0.	0.	97.47	0.	0.	0.	0.	0.	0.	0.	0.	2.53	0.
**P6**	0.	0.	0.	0.	0.	98.52	0.59	0.	0.	0.	0.	0.89	0.	0.	0.
	0.15	0.	0.	0.	0.	95.72	0.	0.	0.	0.	0.	3.69	0.	0.44	0.
**P7**	0.	0.	0.	0.2	0.	0.	99.8	0.	0.	0.	0.	0.	0.	0.	0.
	0.	0.	0.	4.88	0.	0.	93.09	0.	0.	0.	0.	2.03	0.	0.	0.
**P8**	0.	0.	0.	0.	0.	0.	0.	96.98	0.	0.	0.	0.	3.02	0.	0.
	0.	0.	0.	0.	0.	0.	0.	92.58	0.	0.	0.	0.	7.42	0.	0.
**P9**	0.	0.	0.	0.	0.	0.	0.	0.	91.53	0.	6.88	0.	0.	1.59	0.
	0.	0.	0.	0.	0.	0.	0.	0.	97.62	0.	2.38	0.	0.	0.	0.
**P10**	0.	0.	0.	0.	0.	0.	0.	0.	0.	100.	0.	0.	0.	0.	0.
	0.	0.	0.	0.	0.	0.	0.	0.	0.	100.	0.	0.	0.	0.	0.
**P11**	0.	0.	0.	0.	4.3	0.	0.2	0.	0.	0.	95.49	0.	0.	0.	0.
	0.	0.	0.	0.	8.81	0.	1.23	0.	0.	0.	89.55	0.	0.	0.2	0.2
**P12**	0.	0.67	0.	0.	0.	10.72	0.	0.	0.	0.	0.	88.47	0.	0.	0.13
	0.	0.	0.	0.	0.	17.69	0.	0.	0.	0.	0.	82.17	0.	0.	0.13
**P13**	0.	0.	0.	0.	0.	0.	0.	0.	0.	0.	1.79	0.	98.21	0.	0.
	0.	0.	0.	0.	0.	0.	0.	0.32	0.	0.	0.	0.	99.68	0.	0.
**P14**	4.89	0.	0.	0.	3.33	0.	1.11	0.	0.	0.	0.	0.	0.	90.22	0.44
	1.56	0.	0.	0.	0.44	0.	0.	0.	0.22	0.	0.	0.	0.	97.56	0.22
**P15**	0.42	2.51	0.31	0.	0.	0.	0.	2.93	0.	0.1	0.	0.52	1.26	0.	91.94
	0.	3.56	0.	0.	0.	0.	0.73	0.	0.	0.	0.	1.99	0.	0.	93.72

**Table 5:** Comparison with the related works.

	Environment	Objectiveness	Pilots	Signals	UAV	Extensible	Accuracy
Soufan (2017) [11]	Robotic lab	Authentication	5	Pilot instructions	quadcopter	No	82%
Soufan (2018) [12]		Identification	20				89%
Alkadi (2021) [13]		Authentication	20	Pilot instructions motion sensors		Yes	97%
Ours	Controlled & Natural environment	Identification	15	uORB topics			95.71%

**Table 6:** Comparison with the most related works on P450 in nature and constrained environment.

	T1	T2	T3	T4	T5	T6	T7	T8	T9	T10	T11	T12	T13	T14	T15
**QD** [12]	42.14	57.09	93.72	93.99	86.74	79.36	59.63	95.48	59.07	46.11	63.52	72.81	43.66	45.84	22.72
	45.32	63.98	90.47	87.71	75.43	82.11	68.55	90.53	49.67	51.92	60.34	69.17	60.22	58.11	39.73
**RF** [12]	29.82	85.69	49.13	93.48	71.08	51.32	45.49	92.37	89.87	68.88	79.67	53.97	79.87	72.74	58.74
	35.77	74.51	45.49	90.32	59.77	59.14	52.88	82.17	80.15	69.87	62.29	55.41	78.32	63.33	51.25
**Bagging** [12]	47.12	20.05	19.58	99.82	61.18	53.17	42.27	80.61	33.86	92.08	76.02	83.19	63.89	23.16	22.26
	40.11	30.79	17.98	80.34	53.27	59.16	38.65	76.44	30.78	82.17	77.29	87.44	62.63	30.35	29.87
**DT** [12]	18.48	66.86	97.11	90.29	81.29	36.51	71.51	27.11	27.42	23.33	47.08	15.89	76.94	48.73	31.62
	23.88	60.64	84.87	75.91	71.17	45.35	82.44	34.47	31.82	38.65	45.22	19.97	49.12	59.88	19.65
**LSTM** [13]	91.73	94.41	92.89	93.32	89.35	95.52	93.22	94.44	93.37	96.53	94.16	95.12	96.16	94.47	96.61
	94.42	91.99	95.25	94.17	93.89	95.11	98.22	93.77	92.29	91.88	94.37	94.41	95.33	94.48	94.37
**Feature** [13]	47.21	68.45	92.73	95.41	89.98	83.67	82.81	79.49	85.62	81.66	76.27	80.35	62.51	74.88	61.67
	55.32	77.45	82.62	85.68	91.98	85.33	92.41	89.55	65.77	84.32	79.31	90.41	83.15	65.97	71.72
**Voted** [13]	58.95	61.26	84.31	93.82	100.	70.45	98.37	68.36	46.03	49.05	41.59	10.98	72.56	66.94	36.18
	60.45	60.57	84.62	94.51	95.27	71.34	98.44	64.52	41.27	49.61	39.85	12.51	71.19	68.48	33.75
**SVM** [23]	76.02	63.17	98.21	100.	90.21	79.38	90.45	99.65	89.94	100.	84.24	56.25	92.93	91.52	90.24
	81.41	73.52	95.35	94.24	85.45	68.97	84.33	93.12	91.47	84.71	87.24	58.12	89.93	93.71	94.14
**XGB** [23]	93.72	92.75	95.25	91.88	94.55	70.18	98.32	94.51	94.84	95.57	72.13	89.33	94.71	99.72	95.21
	88.95	94.17	85.93	92.08	99.55	82.18	96.11	93.42	89.88	85.99	82.13	90.16	88.35	94.32	96.17
**RF** [23]	94.76	99.88	96.56	91.92	95.45	80.53	95.21	91.19	92.45	98.06	95.45	50.81	63.82	94.61	95.17
	97.33	92.85	93.71	90.45	93.16	84.33	93.35	93.39	94.17	95.21	94.42	63.22	73.41	91.18	94.57
**Ours**	100.	99.12	88.78	95.54	85.12	99.24	91.22	99.24	89.72	73.54	87.82	89.12	100.	100.	94.72
	97.73	99.43	98.91	99.22	97.36	96.23	98.22	100.	100.	98.32	96.22	100.	100.	99.36	97.92

**Table 7:** Comparison with the most related works on S500 in nature and constrained environments.

	T1	T2	T3	T4	T5	T6	T7	T8	T9	T10	T11	T12	T13	T14	T15
**QD** [12]	22.72	56.73	63.52	93.99	63.29	45.19	35.77	92.51	79.36	93.72	59.63	20.37	67.14	86.44	35.18
	35.22	53.47	60.82	88.31	55.37	42.44	38.95	88.74	81.52	91.33	50.11	19.47	55.25	83.44	39.13
**RF** [12]	60.12	56.61	81.01	91.25	97.89	69.71	93.91	69.38	49.73	49.03	40.61	12.56	72.38	65.53	33.59
	64.24	62.88	85.49	90.37	89.72	73.41	92.33	72.17	50.58	59.45	38.11	15.41	78.32	63.27	41.62
**Bagging** [12]	34.19	52.56	93.33	99.82	86.56	32.27	77.25	24.57	58.64	41.55	27.21	61.92	57.14	34.29	48.48
	46.11	48.47	87.88	90.34	83.55	29.99	68.65	26.14	49.78	52.51	28.49	57.44	62.63	30.35	59.87
**DT** [12]	33.88	20.64	84.87	85.91	84.17	45.35	56.61	74.47	51.82	49.49	35.22	79.31	62.12	63.22	52.65
	45.14	36.51	77.62	92.33	75.98	52.17	67.32	82.13	58.77	48.65	33.11	57.41	82.54	61.17	56.18
**LSTM** [13]	87.73	92.41	88.89	89.22	93.35	94.35	91.77	88.48	93.37	95.21	91.11	89.12	89.97	93.32	91.44
	89.45	93.02	85.17	91.44	97.02	91.38	88.53	91.92	94.44	93.38	85.61	77.38	88.47	95.19	92.03
**Feature** [13]	55.13	58.41	72.71	95.32	89.98	82.75	77.95	89.41	85.36	91.52	76.27	87.39	91.51	86.47	82.21
	49.33	59.35	63.92	95.41	84.12	73.99	62.37	85.47	76.11	93.37	85.11	82.35	83.44	84.18	81.67
**Voted** [13]	34.39	92.61	49.13	96.36	68.71	47.35	43.64	98.31	97.04	88.62	83.25	63.17	80.35	74.36	57.91
	29.45	89.57	51.62	94.51	75.27	51.34	44.44	84.52	91.27	89.61	79.85	62.51	85.19	68.48	53.75
**SVM** [23]	90.05	85.93	85.36	91.92	92.82	80.68	88.31	98.32	89.02	89.77	98.81	94.56	64.77	78.72	74.97
	86.41	83.17	75.35	94.24	90.21	79.38	90.45	93.62	89.88	94.71	84.24	86.25	61.93	73.71	80.24
**XGB** [23]	93.34	92.21	91.17	92.04	89.88	98.67	68.85	96.31	65.82	96.61	94.13	92.52	60.38	93.32	93.19
	93.72	92.75	95.25	91.88	94.55	70.18	98.32	94.51	94.84	95.57	72.13	89.33	94.71	99.72	95.21
**RF** [23]	91.95	93.37	90.44	96.12	89.03	98.41	36.68	100.	75.11	96.66	96.41	88.79	99.51	100.	97.38
	92.37	90.48	93.71	91.92	95.45	80.53	95.21	91.19	92.45	98.06	95.45	50.81	63.82	94.61	95.17
**Ours**	100.	91.54	88.49	100.	91.98	98.52	99.81	96.98	91.53	100.	95.49	88.47	98.21	90.22	91.94
	94.56	96.07	91.48	100.	97.47	95.72	93.09	92.58	97.62	100.	89.55	82.17	99.68	97.56	93.72

**Table 8:** Compared to the standard algorithms with different incremental steps.

	7	8	9	10	11	12	13	14	15
lwf [32]	97.58	93.44	89.26	83.12	76.45	72.22	68.27	64.66	61.25
	96.41	-	89.33	-	81.12	-	76.19	-	64.25
	97.33	-	-	-	83.39	-	-	-	65.58
ewc [29]	98.22	95.36	92.41	88.99	85.68	84.44	83.39	83.22	81.08
	99.18	-	93.46	-	87.75	-	84.44	-	82.08
	97.32	-	-	-	85.59	-	-	-	83.14
mas [30]	98.11	95.45	88.67	85.44	82.21	74.65	69.91	65.44	60.12
	97.26	-	86.12	-	75.34	-	67.22	-	63.72
	97.12	-	-	-	79.11	-	-	-	64.41
ours	98.02	98.76	98.02	97.51	95.52	94.49	94.22	93.98	92.94
	99.02	-	97.33	-	94.46	-	93.18	-	93.18
	97.94	-	-	-	95.67	-	-	-	92.94

## Data Availability

Data are unavailable due to privacy.

## Abbreviations

The following abbreviations are used in this manuscript:

UAV	Unmanned Aerial Vehicle
GPS	Global Positioning System
GCS	Ground Control Station
MEMS	Micro-Electro-Mechanical Systems
LD	Linear dichroism
QD	quadratic discriminant
SVM	Support vector machine
kNN	K-nearest neighbors
RF	Random forest
DT	Decision tree
LSTM	Long short-term memory
SGD	Stochastic gradient descent
ESC	Electronic Speed Controller
NED	North East Down
ReLU	Rectification Linear function
lwf	learning without forget
ewc	elastic weight consolidation
mas	memory aware synapses
SOTA	state-of-the-art work
